# Training and Experience of Peer Reviewers: An Additional Variable to Consider

**DOI:** 10.1371/journal.pmed.0040143

**Published:** 2007-03-27

**Authors:** Erik Kulstad

I read with interest your article [1] on peer reviewers' review quality and the relationship to previous training and experience, particularly the fact that your study found no easily identifiable types of formal training or experience that predict reviewer performance. As you conclude, without a better understanding of the skills in scientific peer review, journals and editors will have difficulty in systematically improving their selection of reviewers.

I wonder if an additional variable not examined in your study may prove potentially predictive of performance, namely the time committed by a reviewer to the review process in general, or a given review in particular. This data point could easily be provided by a reviewer, albeit with the caution that a self-reported number will have some subjectivity that is immeasurable. This variable could be specified either as the a priori time that an individual reviewer is willing and/or able to put towards completing a review or a self-reported time spent in actually completing a review. Either a simple dichotomized variable, say, less than four hours or greater than four hours spent on a review, or a measurement on a continuous scale, might be revealing.

Perhaps if the self-reported time commitment to a given manuscript review is found to correlate with quality of the review in a univariable or multivariable model, an additional criteria for selecting reviewers can be based on this question. A positive correlation might also explain the paradoxical findings of worse performance when being a peer reviewer for another journal or when serving on an institutional review board. Increased availability of time to commit to the process may also explain the findings of improved review quality with younger training status, if one presumes that free time diminishes with age!
